# Downregulation of Matrix Metalloproteinases 2 and 9 is Involved in the Protective Effect of Trehalose on Spinal Cord Injury

**DOI:** 10.22088/IJMCM.BUMS.7.1.8

**Published:** 2018-03-23

**Authors:** Masoumeh Mirzaie, Mehrnaz Karimi, Hossein Fallah, Mohammad Khaksari, Mahdieh Nazari-Robati

**Affiliations:** 1 *Department of Clinical Biochemistry, Afzalipour School of Medicine, * *Kerman* * University of Medical Sciences, * *Kerman* *, Iran.*; 2 *Neuroscience Research Center, Institute of Neuropharmacology, Kerman University of Medical Sciences, Kerman, Iran.*; 3 *Endocrinology* *and Metabolism Research Center,* *Institute* *of Basic and Clinical Physiology Sciences, Kerman University of Medical Sciences, Kerman, Iran.*

**Keywords:** Spinal cord injury, trehalose, matrix metalloproteinases

## Abstract

Upregulation of matrix metalloproteinases (MMPs), in particular *MMP-2* and *MMP-9* contributes to secondary pathogenesis of spinal cord injury (SCI) via promoting inflammation. Recently, we have reported that trehalose suppresses inflammatory responses following SCI. Therefore, we investigated the effect of trehalose on *MMP-2* and *MMP-9* expression in SCI. A weight-drop contusion SCI was induced in male rats. Then, the animals received trehalose at three doses of 10 (T10), 100 (T100) and 1000 (T1000) mM intrathecally.* MMP-2* and *MMP-9* transcripts were then measured in damaged spinal cord at 1, 3 and 7 days after trauma, and compared with vehicle and sham groups. Additionally, behavioral analysis was conducted for 1 week using Basso-Beattie-Bresnahan (BBB) locomotor rating scale. Our data showed an early upregulation of *MMP-9* at 1 day post-SCI. However, *MMP-2* expression was increased at 3 days after trauma. Treatment with 10 mM trehalose significantly reduced *MMP-2* expression in 3 and 7 days (P< 0.01) and *MMP-9* expression in 1, 3, and 7 days (P< 0.05) post-damage compared with vehicle. Nonetheless, downregulation of both* MMPs* was not observed in T100 and T1000 groups. In addition, T10 group showed more rapid recovery of hind limb strength compared with T100 and T1000 groups. We propose that the neuroprotective effect of low dose trehalose is mediated by attenuation of *MMP-2* and *MMP-9* expression.

Spinal cord injury (SCI) is a major public health problem leaving the patients with life-long disabilities. In SCI, the initial mechanical impaction on the spine leads to a neurological damage that is named “primary injury”. The mechanical damage causes a complex cascade of biological changes, which is known as “secondary injury”. This injury occurs days to years after SCI, and leads to further neurological deficits. Secondary injuries include oxidative stress, inflammation, immune responses, changes in the expression of receptors and ion channels (1, 2).

Oxidative stress and inflammation play a critical role in SCI pathogenesis. It has been shown that reactive oxygen and nitrogen species, including nitric oxide and hypochloric acid, as well as powerful inflammatory mediators such as IL-1β and TNF-α regulate the amount of matrix metalloproteinases (MMPs). MMPs are a large family of zinc-bound extracellular proteases that contribute to digestion of extracellular matrix components as well as some of cell surface proteins. In spinal cord, MMPs break down laminar components, and lead to the destruction of blood-spinal cord barrier (3). MMP-2 and MMP-9, which are also named gelatinase A and B respectively, are of great importance in SCI. *MMP-2* and *MMP-9* are mainly expressed in activated astrocytes after SCI, and inhibition of their activity has protective effects on neuronal cells (4, 5).

The efficacy of several compounds in treatment of SCI has been demonstrated to be through their effects on MMPs. An example is the melatonin which reduces the amount of MMP-2, MMP-9, and some of oxidative stress and inflammatory factors in SCI (6). Furthermore, fluoxetine was shown to decrease the level of inducible nitric oxide synthase (iNOS) and MMP-2 and therefore, reduced the destruction of blood spinal cord barrier in an animal model of SCI (7). It was also shown that fenofibrate elevated neurological recovery by exerting anti-inflammatory effect evidenced by a decrease in *iNOS*, *COX2* and *MMP-9* expression (8). Another compound that appears to have anti-inflammatory and anti-oxidant properties is trehalose. Trehalose is a non-reducing disaccharide that occurs naturally in many living organisms, including plants, insects, fungi, and bacteria. Trehalose has been shown to inhibit inflammation in endotoxin shock. In addition, trehalose suppresses inflammation, oxidative stress, and vasospasm due to subarachnoid hemorrhage. Furthermore, trehalose inhibits inflammatory and proteolytic activity of MMP-9, and decreases *iNOS* expression in macrophages (9-11).

Regarding anti-inflammatory and anti-oxidant properties of trehalose and the effect of inflammation and oxidative stress on activation of MMPs, and considering the role of MMPs in secondary damage after SCI, this study was conducted to investigate the effect of trehalose on expression of *MMP-2* and *MMP-9* in an animal model of SCI.

## Materials and methods

Animals

A total of 90 male Wistar rats weighing 250-300 g were tested in 6 groups in three different times (n=5 in each group). Sham group: rats were subjected to laminectomy; SCI group rats were subjected to both laminectomy and spinal cord injury; vehicle group rats received 6 μl of phosphate buffered saline (PBS) to the lesion site immediately after SCI; trehalose 10, 100 and 1000 groups rats received trehalose at 10, 100 and 1000 mM concentrations in a volume of 6 μl intrathecally following SCI.

**Spinal cord injury**

All expriments were approved by the Ethics Committee of Kerman University of Medical Sciences (IR.KMU.REC.96-3), and performed according to the guide for the care and use of laboratory animals by National Institutes of Health (NIH).

To create a lesion, animals were first anesthetized with intraperitoneal injection of ketamine (50 mg/kg) and xylazine (5 mg/kg). SCI was made by dropping a 10 g rod from a distance of 2.5 cm onto the spinal cord at T9-T10 level. After injection of PBS and trehalose based on the groups mentioned, muscles and skin were sutured on the site of lesion, and disinfected. Then, animals were placed in a warm room until recovery from anesthesia. Rats were then housed in a temperature-controlled room with alternating 12 h light and dark cycles and adequate access to food and water. Animal bladders were manually voided twice a day until normal bladder function returned. To prevent infection, gentamycin (12 mg/kg) was administered every other day. Then, rats were killed at 1, 3, and 7 days following SCI. After removing one cm of spinal cord with a lesion in the middle, spinal cord tissues were immediately frozen in liquid nitrogen and stored at -70 °C.

**Locomotor function**

Locomotor recovery was assessed using an open-field testing paradigm, the Basso-Beattie-Bresnahan (BBB) locomotor rating scale, which is based on a 21-point-scale developed originally in spinal cord injured rats (12). This scale assesses 10 distinct categories that range from limb movement to tail position, and involves detailed observations of joint, movement, stepping, and coordination. In this experiment, each animal was tested in an open field for 5 minutes every day until day 7 post-injury.Uninjured animals exhibited a locomotor score of 21, whereas animals that showed complete hindlimb paralysis were scored as 0.

**RNA extraction and Real-time PCR**

Spinal cords were homogenized in Trizol reagent using a homogenizer. Total RNA of spinal cord tissues was extracted according to the manufacturer’s protocol. The concentration and purity of RNA were determined by calculating the ratio of absorbance at 260 and 280 nm. Then, 0.5 μg of total RNA was transcribed into cDNA using Takara cDNA synthesis Kit. cDNA amplification and detection were run on a StepOnePlus (Applied Biosystems) real time PCR system. All reactions were performed using 2x qPCR master mix (Ampliqon, Odense, Denmark), gene specific primers (5 μM), and approximately 50 ng cDNA in 20 μl total volume. The sequences of primers were as follows: *MMP-2*, forward: 5'-AGCTCCCGGAAAAGATTGAT-3' and reverse: 5'-TCCAGTTAAAGGCAGCGTCT-3'; *MMP-9*, forward: 5'-TCGCTCGGATGGTTATCGC-3' and reverse: 5'-AAGACGCACATCTCTCCTGC-3'; *GAPDH*, forward: 5'-AACCCATCACCATCTTC-CAG-3' and reverse: 5'-GTGGTTCACACCCATC-ACAA-3'. Primers efficiency were determined by generating a standard curve for *GAPDH*, *MMP-2*, and *MMP-9* prior to performing the assay on samples. PCR conditions were 40 cycles of 95 °C for 30 s, 62 °C for 30 s, and 72 °C for 30 s. The specificity of amplification was verified by analyzing melting curves and subsequent gel electrophoresis. *GAPDH* was used as an internal control. Then, relative gene expression was normalized to *GAPDH* and calculated using ΔΔCT method (13).

**Statistical analysis**

Non-parametric Kruskal-Wallis test was used to determine differences among group means followed by Mann-Whitney analysis. Data were expressed as mean ± SEM. A preset value of P< 0.05 was considered statistically significant.

## Results

**Fig. 1:**
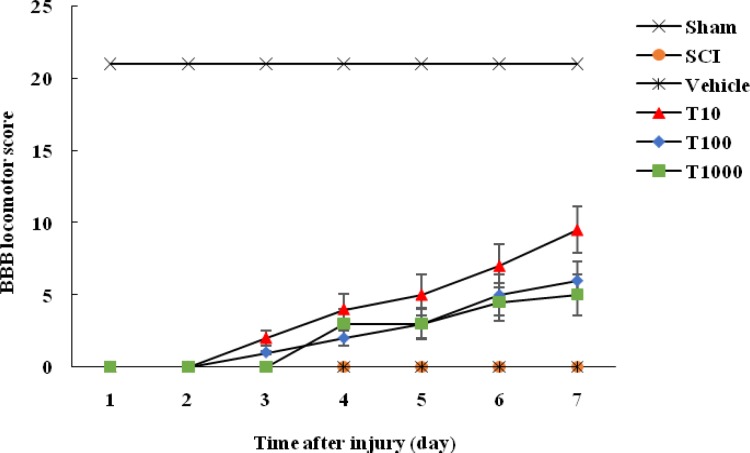
Effect of trehalose on BBB locomotor score. Injured rats were subjected to intrathecal injection of trehalose at doses of 10, 100 and 1000 mM. Locomotor function was measured for one week using BBB rating scale. Data were presented as mean ± SEM

Evaluation of locomotor function demon-strated that BBB score in sham group was 21 every time point throughout the experiment. Nevertheless, after the surgery, BBB score of other groups was approximately 0, which indicated the success of SCI model establishment. In addition, hindlimb motor function was not improved in SCI and vehicle groups at various time points. However, BBB score was significantly increased in trehalose treated groups compared with vehicle group on day 7 after damage (*P*< 0.01) which indicated recovery of locomotor function in trehalose treated animals. In addition, BBB scores of rats treated with 10 mM trehalose tended to be higher than 100 and 1000 mM trehalose treated groups at 7 days post-injury (*P*< 0.05). Therefore, the rats treated with 10 mM trehalose exhibited more rapid recovery of hindlimb motor function as represented by higher BBB score (Figure 1). 

The expression level of *MMP-9* and *MMP-2* as potential target genes was quantified in 1, 3, and 7 days after injury using real-time PCR. At each time point, *MMP-9* mRNA level in sham group was significantly lower than SCI group (P< 0.05). Therefore, SCI led to increase in *MMP-9* mRNA level which reached to a peak in 3 days post-injury. Although, the level of *MMP-9* mRNA between vehicle and SCI groups showed no significant difference at any time points (*P*>0.05). Therefore, PBS did not have effect on *MMP-9* expression level.

**Fig. 2:**
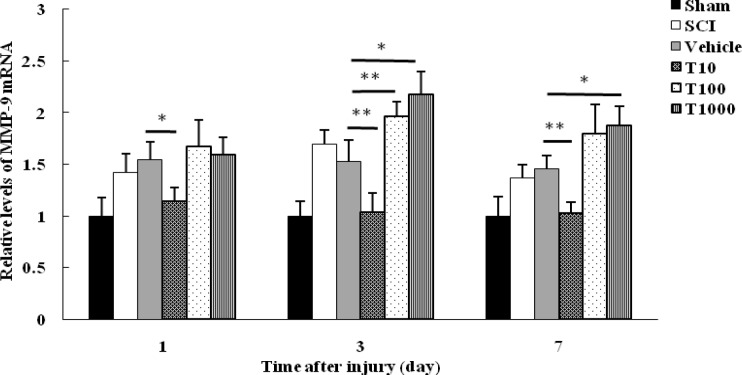
Effect of trehalose on *MMP-9* mRNA level. Spinal cord mRNA level was measured in different groups at 1, 3, and 7 days following SCI. Values were standardized against *GAPDH*. The results were presented as fold change of sham. Data were expressed as mean   ±  ±  SEM. * and ** represent P  0.05 and P  0.01, respectively versus vehicle group

At 1, 3, and 7 days following trauma, *MMP-9* mRNA level in T10 group significantly decreased compared with vehicle group (*P*< 0.05 on day 1; P< 0.01 on days 3 and 7). However, *MMP-9* mRNA level in T100 and T1000 groups remained unchanged at 1 day following trauma compared with vehicle group (P>0.05). *MMP-9* expression in T100 group was upregulated at 3 and 7 days post-injury which was significant at 3 days after SCI in comparison with vehicle group (*P*<0.01). Furthermore, treatment with 1000 mM trehalose enhanced significantly *MMP-9* expression at 3 and 7 days after trauma in comparison with vehicle group (*P*< 0.05) (Figure 2).

**Fig. 3:**
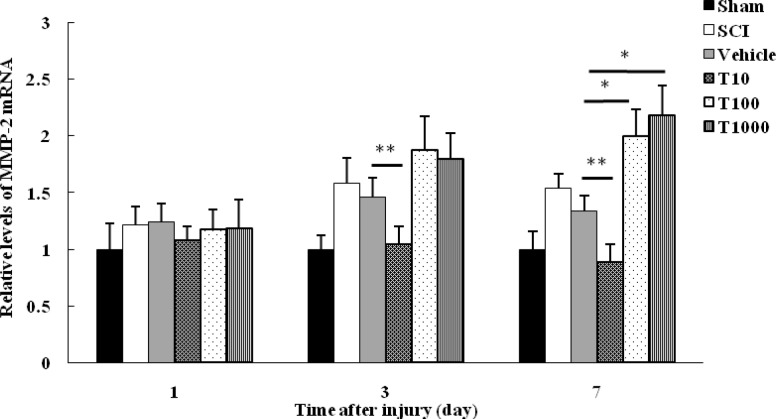
Effect of trehalose on *MMP-2* mRNA level. Spinal cord mRNA level was measured in different groups at 1, 3, and 7 days following SCI. Values were standardized against *GAPDH*. The results were presented as fold change of sham. Data were expressed as mean   ±  ±  SEM. * and ** represent P  0.05 and P  0.01, respectively versus vehicle group

No significant difference was observed in *MMP-2* mRNA level between sham and other groups at 1 day following injury. yet, at 3 and 7 days post-trauma, *MMP-2* mRNA level in sham group was significantly lower in comparison with SCI group. In SCI group, *MMP-2* expression was upregulated at 3 days, and remained elevated at 7 days post-SCI. Similar to *MMP-9*, the level of *MMP-2* mRNA in vehicle group was not significantly different in comparison with SCI group at various time points (P> 0.05). In addition, there was no significant difference in mRNA level of *MMP-2* between trehalose treated groups and vehicle group at 1 day post-trauma (P> 0.05). Treatment with 10 mM trehalose significantly downregulated* MMP-2* expression at 3 and 7 days post-SCI in comparison with vehicle group (*P*< 0.01). Despite that, *MMP-2* was upregulated following treatment with 100 and 1000 mM trehalose at 3 and 7 days post-injury which was significant at 7 days after trauma when compared with vehicle group (*P*< 0.05) (Figure 3).

Evaluating *MMP-9* and *MMP-2* expression revealed prominent *MMP-9* mRNA level by 3 days post-injury followed by a gradual rise in *MMP-2* by 7 days after damage. These findings highlight a unique profile for *MMP-9* and *MMP-2*, with the first one dominating in the more acute phase, and the second one being associated with a delay post-trauma.

## Discussion

MMPs are found in a variety of cells, and exist in a latent form (zymogen) until activated by exogenous stimuli or pathological conditions. MMPs play important roles in inflammatory responses, and tissue remodeling associated with various pathological conditions including cerebral ischemia, neurodegenerative diseases, and head trauma (6). In brain and spinal cord injury, MMPs degrade components of basal lamina, leading to disruption of blood-brain-barrier (BBB), and increase oxidative stress, demyelination, leukocyte trafficking, edema, and hemorrhage (3).

Our study demonstrated that *MMP-2* and *MMP-9* mRNA level were upregulated in SCI. *MMP-2* and *MMP-9* have constitutive expression in uninjured states. However, in injured spinal cord, mechanical stimuli, reactive oxygen species, as well as powerful inflammatory mediators such as IL-1β and TNF-α cause a direct elevation in MMPs (14). We previously showed that IL-1β and TNF-α level increased in an animal model of contusion SCI which may stimulate *MMPs* gene expression (15). Our finding showed upregulation of *MMP-9* mRNA level at 1 day after SCI which peaked at 3 days, and declined at 7 days post-trauma. In contrast, *MMP-2* demonstrated delayed upregulation on day 3 which persisted until 7 days post-injury. The expression profile of *MMPs* was reported by evaluation of mRNA transcripts in other studies. Upregulation of *MMP-9*, *MMP-3,* and *MMP-7* mRNA transcripts was observed within 24 h after injury whereas increased expression of *MMP-2*, *MMP-12*, and *MMP-13* was delayed until 5 days after trauma in a murine model of SCI (16). At protein level, gelatin zymography assay defined that the predominant gelatinase activity in injured spinal cord was due to MMP-2 and MMP-9. It also showed a remarkable switch from the early expression of *MMP-9* to *MMP-2* at 7 days post-injury (17).

Aberrant expression of *MMPs* is involved in other CNS disorders including stroke and brain trauma (18). The involvement of MMPs in blood brain or blood-spinal cord barrier disruption facilitates immune cells infiltration, increases tissue damage, induces apoptosis, and eventually impairs functional recovery after injury (19, 20). Here, our results showed that SCI led to functional impairment in all groups evidenced by BBB scores.

The present study documented that low dose trehalose exerts protective effects in SCI through reducing *MMP-2* and *MMP-9* mRNA level. Trehalose is a non-reducing disaccharide which is widely distributed in nature (21). It protects cells during exposure to a range of environmental stresses including heat shock, dehydration, and hypoxia (22). In addition, several studies have shown that trehalose inhibits the inflammatory cascade that in turn leads to oxidative stress and cytokines production (23, 24). Various combinations of pro-inflammatory molecules as well as free radicals induce MMPs, especially MMP-9 activity (25, 26). We previously showed that trehalose minimizes oxidative damage and inflammatory cytokines in SCI (unpublished data). Therefore, reduction in *MMP-2* and *MMP-9 *mRNA levels by trehalose may most likely be attributed to inhibition of IL-1β and TNF-α. Esposito et al. demonstrated that melatonin which attenuates TNF-α production in SCI confers a significant downregulation of MMP-9 and a modest depression of MMP-2 activity (6). Similarly, the report by Mao et al. showed that reduced *MMP-9* expression in injured mice treated with sulforaphane was associated with a decreased level of TNF-α (27).

Our data showed a decrease in both* MMPs* transcripts which occurred with 10 mM trehalose treatment. In spite of that, treatment with 100 and 1000 mM trehalose increased mRNA levels of *MMP-2* and *MMP-9* at 3 and 7 days post-SCI which indicates that trehalose effect occurred in a dose-dependent manner. We speculated that high dose trehalose changed the cellular and molecular environment of lesion likely through increasing osmolality which resulted in different transcriptional regulation of *MMP-2* and *MMP-9*.

Upregulation of *MMP-2* and *MMP-9* has been implicated in secondary damage after SCI through degradation of basal components of blood spinal cord barrier (BSCB), and subsequent inflammatory events (28). Jang et al. demonstrated that photothrombotic SCI increased MMP-9 level as well as water content of spinal cord due to BSCB disruption (29). However, treatment with 17β-estradiol inhibited *MMP-9* expression, and thereby attenuated BSCB disruption and hemorrhage after SCI (30). Similar results were achieved following treatment with valproic acid in a rat model of ischemia (31). Our results suggested that neuroprotective effects of trehalose were mediated in part by reducing *MMP-2* and *MMP-9* expression, and likely by attenuation of BSCB permeability. Hence, further study is required to elucidate the effect of trehalose on BSCB disruption after SCI.

Upregulation of *MMP-2* and *MMP-9* contributes to apoptosis in both neurons and glial cells likely through increase in BSCB permeability, and destructive inflammatory responses. As a consequece, inhibition of MMP-2/MMP-9 activity leads to significant reduction in apoptosis (32, 33). Lee et al. showed that valproic acid improves functional recovery after SCI via inhibition of MMP-9 activity, inflammatory mediators expression, and cell apoptosis (31). In addition, Hong et al. indicated that treatment with N-(2-chloroethyl)-eicosatetraenamide inhibits MMP-2 and MMP-9 activity and promotes functional improvement in a mouse compression model of SCI (34). Furthermore, beneficial effect of dexmedetomidine on the recovery of motor function after injury to spinal cord was due to *MMP-9* expression suppression and BSCB stabilization (35). Moreover, functional recovery was observed following treatment with ethanol extract of *Bupleurum falcatum* which inhibits inflammation, and attenuates *MMP-2* and *MMP-9* expression and activation (36). Consequently, we believe that reduction in *MMP-2* and *MMP-9* mRNA level after low dose trehalose treatment may contribute to remarkable hindlimb function improvement. Interestingly, a recent study has demonstrated that oral trehalose administration improves functional outcomes following traumatic brain injury. Though, the exact mechanism of its action was not explained (37).

It is important to mention the limitations of our study. In general, MMPs are regulated at transcriptional level, and by post-transcriptional modification. However, in this study, MMP-2 and MMP-9 were measured at mRNA level which is not necessarily predictive of protein and activity level. Additionally, MMPs are modulated by physiological inhibitors, such as tissue inhibitors of matrix metalloproteinases (TIMPs). Upregulation of TIMPs exhibited a neuroprotective function in ischemia damage, BBB disruption, and neuronal apoptosis (38). As a result, TIMPs neuroprotective effect might be of particular significance in SCI treatment which was not measured in this investigation. Furthermore, trehalose potential to regulate BSCB disruption and edema through *MMPs* expression reduction should be investigated in future studies.

In conclusion, this study demonstrated the upregulation of *MMP-2* and *MMP-9* mRNA levels after SCI. Treatment with low dose trehalose successfully decreased mRNA transcripts of both* MMPs* and therefore, improved the functional outcome. Thus, this study suggests a potential therapeutic effect of trehalose in reducing secondary damage following SCI 
